# Point-of-care Diagnostic Tools to Detect Circulating MicroRNAS as Biomarkers of Disease

**DOI:** 10.3390/s140509117

**Published:** 2014-05-22

**Authors:** Luis Vaca

**Affiliations:** Instituto de Fisiología Celular, Universidad Nacional Autónoma de México, Ciudad Universitaria, DF 04510, Mexico; E-Mail: lvaca@ifc.unam.mx; Tel.: +52-555-622-5654

**Keywords:** microRNAs, exosomes, fluids, polymerase chain reaction (PCR), microarrays, diagnostics

## Abstract

MicroRNAs or miRNAs are a form of small non-coding RNAs (ncRNAs) of 19–22 nucleotides in length in their mature form. miRNAs are transcribed in the nucleus of all cells from large precursors, many of which have several kilobases in length. Originally identified as intracellular modulators of protein synthesis via posttranscriptional gene silencing, more recently it has been found that miRNAs can travel in extracellular human fluids inside specialized vesicles known as exosomes. We will be referring to this miRNAs as circulating microRNAs. More interestingly, the miRNA content inside exosomes changes during pathological events. In the present review we analyze the literature about circulating miRNAs and their possible use as biomarkers. Furthermore, we explore their future in point-of-care (POC) diagnostics and provide an example of a portable POC apparatus useful in the detection of circulating miRNAs.

## 1. Introduction

### 1.1. What Are MicroRNAs?

MicroRNAs or miRNAs are a form of small non-coding RNAs (ncRNAs) formed by 19–22 nucleotides in their mature form. MiRNAs are transcribed in the nucleus of all cells from large precursors, many of which have several kilobases in length. They were first identified in 1993 by the Ambros group while studying *C. elegans* development [1]. They have now been found in all animals and plants studied to date, representing about 4% of the genes. The latest count lists over 30,000 miRNAs in humans (miRBase). By binding to a target mRNA at its 3′ UTR (untranslated region), miRNAs mediate post-transcriptional gene silencing in cells via the RNA interference (RNAi) pathway [2]. It is a well established mechanism of post-transcriptional gene regulation, differing from classic epigenetic mechanisms of gene silencing [3].

Extensive literature correlates particular miRNAs with the onset and development of different pathologies, positioning them as possible biomarkers [4]. More recently it has been shown that many different cells and tissues secrete miRNAs in specialized vesicles known as exosomes [5]. The fact that exosomes can travel great distances in the bloodstream and may fuse its content to other cells suggests that miRNAs may function as messengers [5]. Indeed exosomes and even free miRNAs (associated to Ago2) have been isolated from saliva, urine, blood and many other extracellular human and animal fluids [6].

In the present manuscript the author will revise the current literature positioning miRNAs as biomarkers, discuss about the advantages and disadvantages of using this small non-coding RNAs as auxiliary tools in the diagnosis and prognosis of disease.

### 1.2. How Are miRNAs Produced?

As mentioned earlier, miRNAs are generated in the nucleus of all cells from high molecular weight precursors. The processing and maturation of miRNAs is a complex, highly regulated mechanism, involving over a dozen proteins and several steps.

MiRNAs are divided in groups according to their location in the genome. A group includes the miRNAs present in intergenic regions. A second group contains those miRNAs present inside genes (either in introns or exons). miRNAs in the first group have their own promoters and regulatory elements. These miRNAs are transcribed independently of any gene. For the second group, transcription of the miRNA is coupled to the transcription of the gene, using the same promoter. In this second group the transcription and splicing of the mRNA are tightly associated, and during the later the miRNA is generated by a poorly understood maturation mechanism.

The initially transcribed miRNA is generally identified as a pri-miRNA. This primary miRNA suffers a processing consisting in the excision of a hairpin-like structure, which is usually named pre-miRNA. This precursor has a length between 60–80 nucleotides and generates (as mentioned above) a hairpin structure. A type 3 RNAase known as Drosha (Drosha in the fruit fly *Drosophila*, RNASEN (Ribonuclease 3) in humans), or the microprocessor complex conducts the excision of the hairpin structure. Typical cleavage conducted by type 3 RNAases (such as Drosha) leave overhangs at the end of the pre-miRNA, which are later recognized by a GTPase-dependent Ran-Exp5 complex which transports the pre-miRNAs out of the nucleus and into the cell cytosol.

Once in the cytosol, a second type 3 RNAase known as Dicer recognizes the hairpin-like structure of the pre-miRNA. Dicer removes the loop from the hairpin, leaving a double stranded RNA molecule (dsRNA) formed of both the mature miRNA (or leader strand) and the so-called passenger strand (also known as star sequence or miR*). The later is degraded once the dsRNA is loaded into the RNA-induced silencing complex (RISC) [7]. The RISC is the macromolecular protein complex in charge of identifying the mRNA that will be modulated by the miRNA. In the RISC the protein Ago2 (also known as slicer) scans the binding between the mature miRNA and the mRNA for Watson-Crick base paring between the two molecules [8]. If a match is found, the RISC sequesters the mRNA associated to the mature miRNA, preventing the translation of the first one [9].

RISC is transferred to RNA non-membranous organelle centers known as processing bodies (P-bodies) [10]. These centers contain RNAases responsible for degrading mRNAs [10].

### 1.3. How miRNAs End Up in Extracellular Fluids?

As mentioned earlier miRNAs are responsible for the down-regulation of protein synthesis, by binding mRNAs and preventing its translation via their sequestration into P-bodies. As such, miRNAs work in the cell cytosol as modulators of protein synthesis (in the phenomenon known as posttranscriptional gene silencing), thus what would they be doing in extracellular fluids?

The identification of specialized vesicles that are selectively secreted by cells and convey signals to adjacent cells or tissues has been around for over 10 years. These vesicles known as exosomes have complex mechanisms for the packaging of its content, transport and exocytosis, and may function as intercellular signaling devices (for a review see [11]).

Exosomes are small (30–300 nm) extracellular vesicles derived from the multivesicular body (MVB) sorting pathway [12]. The MVB is a late endocytic compartment from which exosomes are formed during its fusion with the plasma membrane [12]. Most cells studied to this date produce exosomes, including reticulocytes, epithelial cells, neurons, and tumor cells [13]. Exosomes have been isolated from many fluids such as urine, serum, bile, saliva, and breast milk [14]. Many cells release exosomes constitutively or after a signal, which in many cases involves increments in intracellular calcium [5]. Originally exosomes were considered vesicles derived from apoptotic cells, but recent studies have demonstrated that exosomes are “signaling devices” that promote intercellular communication by shuttling molecules between cells [13]. Among the molecules identified in exosomes are many miRNAs [15].

The first reports identifying miRNAs in fluids may have generated some skepticism due to the possibility that such events may arise from cell lysis [15,16]. Nowadays it is well accepted that miRNAs can travel in exosomes, and even as free molecules (associated to Ago2) in serum, saliva and several other fluids [17].

The formation and release of exosomes involves rearrangement of the cytoskeleton machinery in charge of bringing together opposing membranes before pinching off the membrane connection and releasing the exosomes into the extracellular space [15]. Like in many exocytic pathways, it is proposed that soluble NSF attachment protein receptor (SNARE) complexes are responsible for bringing the membranes together in opposition [18]. The SNARE complex begins assembling at the N-terminal region of the SNARE motifs and proceeds toward the C-termini, while anchoring the interacting proteins in the membranes [19]. The mechanism by which miRNAs are imported into the exosome prior to exocytosis is poorly understood. Recently the heterogeneous nuclear ribonucleoprotein A2B1 (hnRNPA2B1) has been identified as essential for loading miRNAs into exosomes [20]. It is now evident that hnRNPA2B1 binds to motifs present in the miRNA sequence (now known as EXOmotifs) facilitating its loading into the exosome [20]. Exosomes prevent miRNA degradation while traveling in fluids and facilitate entry of miRNAs into the target cell/tissue via vesicular fusion [5,15].

### 1.4. Why miRNAs Are Attractive Biomarkers?

An ideal biomarker is that whose presence or absence shows a very strong correlation with the onset or development of a particular disease. Ideally such biomarker should be relatively easy to detect using very sensitive methods. Proteins have been classically used as biomarkers, however (when compared to nucleic acids) the methods for protein detection are not very sensitive and prone to false positives.

Typically immunoassays such as enzyme linked immunosorbent assay (ELISA) (commonly used for diagnostics) have in the best-case scenario detection limits in the order of a few picograms (pg) per milliliter (mL) [21]. Depending on the molecular weight of the protein studied, this number may reflect the presence of thousands of molecules per mL. In general, most methods used to detect protein content have detection limits on this order or worse.

Because the polymerase chain reaction (PCR) is an amplification method capable of detecting a few molecules, nucleic acids have taken a central role in molecular diagnostics in the last few years [22]. Traditionally, nucleic acid biomarkers have included messenger RNAs (mRNA) associated with a particular disease, or ribosomal RNAs [23]. The fact that several studies have recently identified circulating miRNAs in many extracellular fluids, and that these miRNAs are especially resilient to degradation, position them as ideal biomarkers [24–30].

Recently, we have been able to detect miRNAs in serum of mice having concentrations as low as a few dozen molecules per mL (unpublished observations). This has been accomplished using highly selective primers in combination with real-time PCR studies. Comparing the ability to detect a few molecules versus several thousands per mL as detection limit, clearly positions the detection of nucleic acids as a winner. A plethora of studies conducted over the last four years have identified many miRNAs as biomarkers for different pathologies, including liver disease [31], cardiac afflictions [32,33], diabetes [34], cancer [35,36], kidney alterations [37], neurodegenerative diseases [38,39], allergy and asthma [40] and even as anti-doping biomarkers [41].

Identifying reliable biomarkers has always been the bottleneck in molecular diagnostics, and miRNAs are not exception. However identification of miRNA biomarkers is in its infancy. Many studies are still required to unambiguously identify miRNAs for specific pathologies. What we have learned from all the studies reported to this date is that a single miRNA associated to a specific pathology may exist only in rare occasions. More likely, one should search for a set (signature) of miRNAs altered in a particular disease [39]. Many miRNAs may be altered in pathologies present in a wide variety of diseases, such as inflammation, fibrosis, edema, *etc.* For instance, many liver diseases initiate with inflammation, which later can evolve into fibrosis [31]. A set of miRNAs altered in inflammation may indicate that this process is occurring somewhere in the body, but cannot be used to pinpoint the particular disease or the organ affected.

Another aspect that complicates the reliable identification of miRNA biomarkers of a particular disease is the fact that only a few copies of these non-coding RNAs are present in fluids, forcing the amplification of the signal prior to detection. For this reason PCR is a very popular method for the identification of circulating miRNAs.

Like all molecular diagnostics methods, validation of the procedure, primers, polymerase, cycles, temperatures, etcetera, is essential to reduce or prevent false positive or negative results. Something frequently neglected in molecular diagnostics is the use of positive controls, resulting in many false negatives when the PCR reaction fails.

### 1.5. Methods for the Detection of Circulating miRNAs and Point-of-Care (POC) Diagnostics

In an ideal situation, a regular visit to your family doctor should include molecular scanning for circulating miRNAs as part of your annual checkup. Unfortunately, all the methods currently utilized for the identification of miRNAs require special training in molecular biology techniques, and cannot be performed by a physician or nurse. Thus, the development of a robust simple to use and sensitive methods for molecular diagnostics is a necessity in modern life.

Even though microarrays has been implemented for the identification of miRNAs, they are not very practical as point-of-care diagnostic tools. Firstly there is considerable time and expertise required for sample preparation. Nucleic acids must be labeled with fluorescent indicators, and later purified for their use in microarrays. Secondly, sample preparation; incubation and all the procedures necessary to obtain the result may take several days. Finally, microarrays are worthwhile when exploring entire genomes, but too expensive for the exploration of only a handful of miRNAs.

Recently we have developed a novel microarray platform which utilizes low density microarrays for the combined identification of proteins and nucleic acids [42]. The use of molecular beacons (MB) as detector of the nucleic acids prevents the need for labeling the sample with fluorescent indicators [43]. This implementation reduces sample preparation times and complexity, while allowing the identification of positive signal in real time [42]. The microarray method we have developed takes advantage of total internal reflection fluorescence (TIRF) illumination, which reduces greatly the background signal generated by human fluids such as blood and urine. This is accomplished by exciting only a thin layer (less than 200 nm) above the microarray slide, preventing the excitation of the bulk solution which may contain elements capable of generating autofluorescence signal [42]. Another advantage of the microarray method we developed is the fact that we can get information about mismatches in sequences by studying the association time courses between the MB and the sample [42]. I will show later on, an example of using association time courses in the identification of miRNAs having only one or two different nucleotides in their mature sequence, such as Let7a and Let7b.

Another powerful tool utilized in the identification of miRNAs is real-time PCR (qPCR). A clear advantage of qPCR for miRNA identification is the fact that this is an amplification reaction (unlike microarrays). The main disadvantage is that requires a good understanding of primer design, annealing temperatures and many other technical aspects hard to convey on medical personnel.

**Figure 1.**
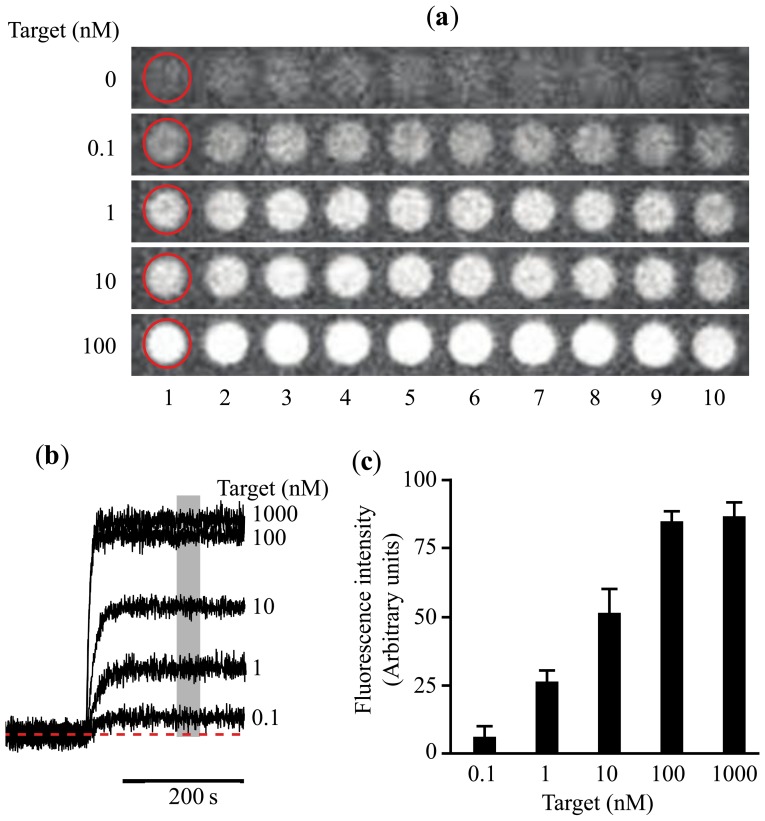
Sensitivity of the total internal reflection fluorescence (TIRF)-based microarray platform. (**a**) Examples of the signal detected by a charge-coupled device (CCD) camera arising from microarray spots developed to detect synthetic DNA sequences (for details please refer to the original publication [42]). To verify the reproducibility of the fluorescent signal the MB was spotted in tandems of 10 (left to right spots). Increasing concentrations of the target sequence were introduced in the system (from 0 used as control, 0.1, 1, 10 and 100 nM). The red circle indicates the region of interest (ROI) from which fluorescence was measured over time; (**b**) Mean fluorescence values obtained from the ROIs on the 10 fluorescent spots in the microarray illustrated in (a). The plots illustrate the time courses of association of the sample to the molecular beacons (MB). Dotted red line shows the background level. Each of the concentrations of the target tested is indicated at the right of the panel; and (**c**) fluorescence intensity (in arbitrary units, AU) illustrating the mean ± standard deviation obtained from the 10 fluorescent spots in the microarray shown in (a). Values were measured at the time indicated by the gray rectangle in (b). Notice that the limit of detection was around 0.1 nM. Figure adapted from [42], with permission from the authors.

Ideally a miRNA detector should be equivalent to a glucose reader. Many years ago glucose determination required specialized equipment, only available in hospitals. Nowadays anyone can purchase a simple glucose reader in the pharmacy for a few dollars. However, developing a portable, easy to use miRNA detector with sufficient selectivity and sensitivity for Point-of-care (POC) diagnostics is not a simple task. Our group has been working for the last five years in the development of a platform useful in POC. Initially we developed a simple microarray assay capable of detecting simultaneously proteins and nucleic acids [42]. However, the detection limit was around 0.1 nM, which is about five times better than the detection limit obtained with immunoassays [44] (Figure 1).

We are currently working on a second version of the system, which will couple an initial PCR reaction with the microarray for detection. This coupled system should provide enough sensitivity to detect a few molecules of miRNA per mL of fluid. The second, equally challenging task, is how to transform this system into a portable device useful for POC. Like many portable devices, we face the challenge of engineering a battery-operated system with sufficient power to run the equipment for several hours. Battery lifetime is a common problem in portable devices, from cameras to mp3 players and tablets.

A portable PCR system requires temperature cycling, which is a major battery drainer. For this reason we began experimenting with isothermal PCR. Isothermal PCR (isoPCR) consists in the use of a DNA polymerase with strand displacement activity, which does not require temperature cycling to separate the double stranded DNA during PCR. isoPCR is a promising technique, already used in molecular diagnostics [45].

Microfluidics is another challenging problem. Transportation of the PCR reaction from the PCR chamber and into the microarray plate in a portable device requires the timely managing of a few microliters over a few millimeters. Dispensing the rinsing solutions and all reagents in a timely fashion complicates the design further.

Finally is the problem of detection. Highly sensitive charge-coupled device (CCD) cameras are big and expensive, requiring cooling and complex electronics. Another possibility is the use of photodiode arrays [46]. Photodiodes are small, very sensitive, and simple to operate photodetectors capable of transforming light into either current or voltage. They can count photons but cannot generate images. Arrays of photodiodes could be engineer to readout the signal generated by the microarray spots. Using large microarray spot areas would help in the amount of signal generated and facilitates the use of one photodiode per microarray spot. This procedure is relatively simple to implement in low-density microarray platforms.

**Figure 2.**
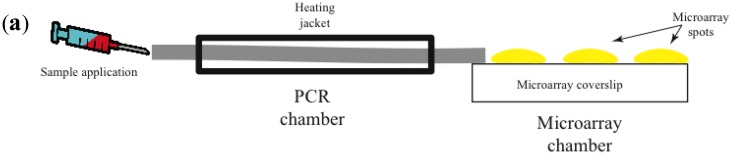

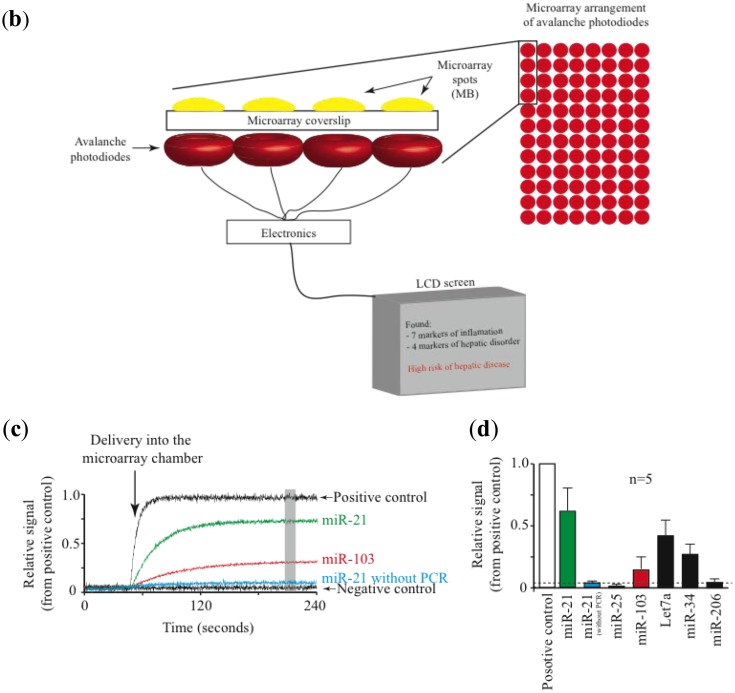
Portable PCR-microarray combined system with potential use in POC diagnostics (**a**) drawing illustrating the dual chamber system, left a needle is used to apply the sample which enters into the PCR (first) chamber. A heating jacket keeps the temperature at 60 degrees Celsius, the temperature at which the Bst DNA polymerase has its highest activity. The PCR chamber contains already a master mix with primers, oligonucleotides and PCR reaction buffers. Sample is maintained in the PCR chamber for 60 min, after which it is transferred into the microarray chamber (right). The microarray chamber contains the coverslip with low-density molecular beacons (MB) printed using a low melting point agarose solution (MB shown in yellow). (**b**) Illustration of the array of avalanche photodiodes arranged to detect fluorescence emitted from the MB in the microarray spots. Photodiodes are connected to an integrated circuit, which reads the current generated by the photons bouncing on the photodiode. The current is then send to a liquid crystal display (LCD) screen for real-time viewing of data and stored on a microSD card for later analysis and plotting. (**c**) Examples of curves illustrating the time courses of relative signal obtained under the different experimental conditions. As positive control an oligonucleotide that matched perfectly to the sequence of a control MB was utilized. For simplicity sake, only the time courses of detection of two miRNAs are depicted (miR-21 in green and miR-103 in red). The blue line illustrates the values obtained applying the same sample but skipping the PCR reaction step (miR-21 without PCR in blue). Notice that signal values are within the noise value. Negative control is a MB for which no matching sequence was present. This value reflects the autofluroescence of the MB alone (without the target present). (**d**) Plots illustrating the mean and standard deviation values obtained from 5 independent reactions for the 6 miRNAs explored in this study (miR-21, miR-25, miR-103, Let7a, miR-34 and miR-206). The dotted line represents the level of autofluroescence (background) signal. Notice that without the PCR amplification step, miR-21 cannot be detected (blue bar). The same sample subjected to the PCR amplification step generates a robust signal for this miRNA (green bar).

Most promising is the use of avalanche photodiodes, which incorporate internal gain via higher electric fields, thus increasing the number of charge carriers that are collected [47]. Another important advantage is that avalanche photodiodes are relatively inexpensive. Figure 2 illustrates a prototype of a combined isothermal PCR-microarray platform for the detection of circulating miRNAs. Sample preparation (outside the device) initiates by the synthesis of complementary DNA (cDNA). Then the cDNA is injected into the device using a Hamilton syringe (Figure 1a). Eventually, this step can be implemented inside the device.

The device has two main chambers: one devoted to isothermal PCR and the second one with the low density microarray printed with molecular beacons (MB), designed to identify mature forms of miRNAs amplified during the PCR reaction (Figure 1a). The microarray chamber consists of an array of avalanche photodiodes positioned under each of the microarray spots (Figure 1b). We manually print large microarray spots using a pipette and MB dissolved in low melting point agarose. The diameter for each microarray spot is about 600 microns. A simple microfluidics system drives the solution from the PCR reaction chamber to the microarray chamber for miRNA identification. The microarray system utilizes our previously described TIRF illumination [42], to reduce background signal arising from the bulk solution.

Initially we engineered a cocktail of primers designed to amplify a set of 6 miRNAs, although the design could be more complex to incorporate more miRNAs in the list. We have explored different isothermal techniques including loop-mediated isothermal amplification (LAMP) [48] and decided that strand-displacement PCR amplification using Bst DNA polymerase is a simpler efficient method [49]. Initial studies have shown that without PCR amplification, the detection of circulating miRNAs using solely the microarray platform is not possible (Figure 1c).

**Figure 3.**
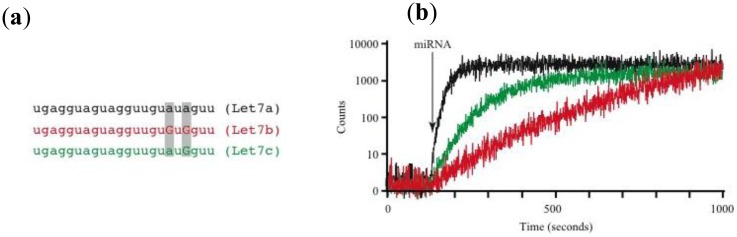
Identification of miRNAs having one or two mismatches with our TIRF-based microarray platform (**a**) mature sequences from Let7a1 (black), Let7b (red) and Let7c (green). Gray rectangles show the nucleotide mismatches found between Let7a1 (used as reference) and Let7b and Let7c; and (**b**) Mean values obtained from the readings of 10 spots (10 independent avalanche photodiodes) for each miRNA tested. The vertical arrow points to the time in which the synthetic miRNA was added to the system (either Let7a1, Let7b or Let7c). All miRNAs were added at a final concentration of 1 nM. Colors of the curves correspond to the sequences in (a). The MB printed on the microarray spots corresponds to the complementary sequence of Let7a1 (perfect matching).

Using this prototype we have identified circulating miRNAs in the serum of mice (Figure 2D). The pattern of miRNA relative abundance is similar to that reported using real time PCR methods [50]. Another important challenge in the detection and identification of circulating miRNAs is the fact that families of closely related miRNAs have only one or few mismatches in their mature sequences. For instance, the Let7 family comprises several related miRNAs (Let7a to Let7i in humans) [51]. Furthermore, Let7a has several isoforms (Let7a1 to Let7a3) [51]. Many Let7 members have several differing nucleotides, but some have only one or two mismatches, such as Let7a1, Let7b and Let7c. Using the association time constants with our microarray method, we have previously shown that it is feasible to identify sequences with one or more mismatches [42]. Figure 3 illustrates an experiment using the device described in Figure 2 and synthetic sequences from Let7a1, Let7b and Let7c. As illustrated in the figure, using the avalanche photodiodes in our microarray platform we found differences in the association time courses for the three aforementioned miRNAs. Not surprisingly, the miRNA showing the slowest association kinetics was Let7b, which has 2 mismatches when compared to let 7a. In this case in particular, the MB printed on the microarrays was designed to match perfectly the Let7a1 sequence. Notice that Let7c, having only one mismatch with Let7a1, showed an intermediate association curve.

Even though, we have shown that in laboratory controlled conditions it is feasible to detect circulating miRNAs in the plasma of living animals with a combined method using an amplification step via PCR and a second identification method based on a novel microarray platform, portable devices like the one reported here will require extensive testing and validation comparing the outcome with more traditional methods of detection, before we can be certain that a device is robust enough for POC diagnostics.

## 2. Experimental Section

### 2.1. Molecular Beacons (MB) Design

All molecular beacons were purchased from Integrated DNA Technologies (Coralville, IA, USA). All MB were designed with HEX as the fluorophore-reporter dye and BHQ1 as the fluorescence quencher. MB were designed to identify 6 miRNAs (miR-21, miR-25, miR-103, Let7a1, miR-34 and miR-206), chosen because they were previously reported in the serum of mice [52]. MB were designed so that four bases from the stem and the remaining 18 bases from the loop matched perfectly the mature miRNAs sequences (Table 1), following the rules previously described [43].

### 2.2. A Portable *polymerase chain reaction* (PCR)-Microarray Combined System

Both PCR and microarray external chambers were produced with acrylic. An internal glass capillary was used to transport the sample between the two chambers. The PCR reaction took place inside the capillary glass. A Peltier module (Digi-key Corporation, Thief River Falls, MN, USA) was used to maintain the temperature around the capillary glass at 60 ± 3 °C. Avalanche photodiodes were purchased from Edmund Optics (Barrington, NJ, USA). Each microarray spot was at the center of every photodiode. To avoid signal contamination from neighboring microarray spots, photodiodes were separated every 5 mm. A simple integrated circuit was produced in house to readout current generated by the photodiodes in a continuous mode. All values were dumped as ASCII code to microSD card for storage and plotted at the same time on the LCD screen. In the figure, data was plotted as relative signal using the positive control as 100% and negative control as 0%. Data was analyzed using Igor pro (Wavemetrics, Lake Oswego, OR, USA).

## 3. Conclusions/Outlook

Circulating miRNAs are here to stay. Abundant experimental evidence indicates that miRNA secretion in exosomes is a highly regulated process, which may be altered during pathological events [15,26,31,34–37,50,53,54]. Thus, circulating miRNA may reflect initial events on the onset of a particular disease or pathology. They may be also utilized as sensors for the progression of disease. The fact that miRNAs are present in exosomes secreted to many different extracellular human fluids provides us with a unique opportunity to develop noninvasive molecular diagnostic tools. One of the main challenges faced by research groups interested in defining particular miRNAs as biomarkers, is finding the right combination of miRNAs present (or absent) in a specific disease, and in a particular extracellular fluid. It is clear from many studies that the miRNAs present in the different extracellular fluids are not the same. There appear to be a selective enrichment of specific miRNAs on any given fluid in normal and sick subjects. The first steps are already on their way, the database of circulating miRNAs was generated recently [55].

For instance, a recent study identified miR-210 as a miRNA upregulated in the plasma from 77 patients with acute kidney injury [56]. Even more, plasma levels of miR-210 could predict patient survival after renal replacement therapy [56]. Interestingly, these patients showed different upregulated miRNAs in urine, with miR-21 and miR-115 showing a clear elevation when compared to healthy volunteers or even patients with other pathologies not related to acute kidney injury.

Other studies have shown the upregulation of miR-122 in both plasma and bile from patients with chronic alcoholism, macrosteatosis and chronic hepatitis C [31]. In this case, the same miRNA was affected in two different fluids. Another essential step is the development of easy to use, selective and highly sensitive methods for the identification of miRNAs in fluids. Current methods (such as PCR and microarrays) require expertise in molecular biology techniques. The development of a method that can be implemented in small clinics and private practice as a routine tool for molecular diagnostics is a key step in generalizing the use of circulating miRNAs as biomarkers. In the present review a prototype combining an amplification step (PCR) and an identification step (microarray), which can be implemented into a portable device has been shown. The combination of PCR and the novel microarray platform we have developed reduces sample preparation and has enough sensitivity to detect a handful of molecules in the plasma. Furthermore, the microarray platform can identify single nucleotide mismatches, as we have previously demonstrated [42]. Nevertheless, much work is required to develop a robust system useful in POC diagnostics as a portable device.

Circulating miRNAs have a great potential, not only in as auxiliary tools in diagnosis, but also to determine if a particular treatment is giving the expected result in fighting a disease, or if it is time to search for alternative therapies. One can even foresee the use of circulating microRNAs as indicators of the sensitivity of a patient to a particular drug, in what is now recognized as individualized medicine. Twenty one years after their identification, miRNAs are still surprising us with interesting possibilities.

## Figures and Tables

**Table 1.** Sequences from the miRNAs used in this study.

**Name**	**Sequence**	**Accession in MiRbase**
miR-21	uagcuuaucagacugauguuga	MI0000569
miR-25	aggcggagacuugggcaauugc	MI0000689
miR-34	uggcagugucuuagcugguugu	MI0000584
miR-103	ggcuucuuuacagugcugccuug	MI0000587
miR-206	uggaauguaaggaagugugugg	MI0000249
Let7a1	ugagguaguagguuguauaguu	MI0000556
